# Herbal Medicine for Pain Management: Efficacy and Drug Interactions

**DOI:** 10.3390/pharmaceutics13020251

**Published:** 2021-02-11

**Authors:** Behdad Jahromi, Iulia Pirvulescu, Kenneth D. Candido, Nebojsa Nick Knezevic

**Affiliations:** 1 Department of Anesthesiology, Advocate Illinois Masonic Medical Center, 836W. Wellington Ave. Suite 4815, Chicago, IL 60657, USA; behdad.jahromi@aah.org (B.J.); iuliappirvulescu@gmail.com (I.P.); kenneth.candido@aah.org (K.D.C.); 2 Department of Anesthesiology, University of Illinois, Chicago, IL 60612, USA; 3 Department of Surgery, University of Illinois, Chicago, IL 60612, USA

**Keywords:** herbal medicine, pain management, herb-drug interactions

## Abstract

Complementary and alternative medicines such as herbal medicines are not currently part of the conventional medical system. As the popularity of and global market for herbal medicine grows among all age groups, with supporting scientific data and clinical trials, specific alternative treatments such as herbal medicine can be reclassified as a practice of conventional medicine. One of the most common conditions for which adults use herbal medicine is pain. However, herbal medicines carry safety concerns and may impact the efficacy of conventional therapies. Unfortunately, mechanisms of action are poorly understood, and their use is unregulated and often underreported to medical professionals. This review aims to compile common and available herbal medicines which can be used as an alternative to or in combination with conventional pain management approaches. Efficacy and safety are assessed through clinical studies on pain relief. Ensuing herb–drug interactions such as cytochrome modulation, additive and synergistic effects, and contraindications are discussed. While self-management has been recognized as part of the overall treatment strategy for patients suffering from chronic pain, it is important for practitioners to be able to also optimize and integrate herbal medicine and, if warranted, other complementary and alternative medicines into their care.

## 1. Introduction

Complementary and alternative medicine (CAM) incorporates a wide range of practices, interventions, therapies, applications, professions, theories, and claims that are not currently part of the conventional medical system. Over time and with supporting scientific data and clinical trials, a specific CAM treatment such as herbal medicine can be reclassified as a practice of conventional medicine. With the present positive social perspective on herbal medicine, its popularity is growing among all age groups [1]. It is a common belief that CAM enables individuals to be more involved with their care, control or offset the adverse events of conventional medicine, and/or find harmony with their culture or philosophies [2]. Patients often seek every possible option for receiving the benefits of medical care while avoiding adverse events [3].

One of the most common conditions in the United States for which adults use CAM is pain. This includes musculoskeletal pain such as cervical, lumbar, or joint pain as well as specific conditions such as arthritis or migraine. Although pain is a physiological and vital response to potential or actual tissue injury, in some cases (such as musculoskeletal pain or special conditions like arthritis) it can become chronic and cause biological changes to the central nervous system or peripheral tissues. Chronic pain can be debilitating and constitutes a high social and economic burden on the health system [4]. In some cases, due to adverse drug reactions, lack of efficacy, or high risk for serious complications, traditional treatments such as opioids or non-steroidal anti-inflammatory drugs (NSAIDs) must be discontinued. Such patients, particularly the elderly, have little option but to suffer from chronic pain or seek nontraditional treatment modalities.

Herbal medicine is one of the most commonly sought forms of CAM. In the United States, herbal medicine is currently used by nearly twenty million Americans [5], with an annual turnover of more than 1.5 billion dollars and growth of approximately 25% each year [6]. According to Hexa Research, the global herbal medicine market was valued at USD 71.19 billion in 2016 [7]. It has been estimated that at least 60% of individuals with arthritis pain or other musculoskeletal pain have tried CAM [8].

According to the World Health Organization (WHO) 1996 guidelines, herbal medicine comprises active end products that contain underground or aerial parts of either plants or plant materials or a combination of both. Most herbal medicines affect the eicosanoid metabolism by inhibiting either both or one of the lipoxygenase and cyclooxygenase (COX) pathways [6]. Their use is generally based on traditional methods, and the ideal extract dose and treatment duration for most herbal medicines have yet to be determined.

Many consumers of herbal medicine believe these treatments are natural and safe, yet herbal medicines contain pharmacological active ingredients that can be associated with numerous and diverse adverse events [6]. Herbal medicines are frequently taken alongside synthetic drugs, which can lead to harmful herb–drug interaction. In many countries, herbal medicines are largely unregulated and have suboptimal product quality; in some Asian herbal mixtures, toxic amounts of heavy metals or mixed synthetic prescription drugs have been reported [6]. Such concerns represent serious safety issues and suggest the possibility of adverse health events for users.

Herbal medicines are usually not the most potent analgesic treatments available. However, they can be highly beneficial for mild to moderate pain [6]. To translate complementary and integrative medicine using herbs into clinical practice and to enable acceptance into treatment guidelines, further rigorous studies are required to confirm the effectiveness and safety of these medicines. The following review aims to consolidate efficacy and safety data for some of the most commonly used herbal medicine for pain relief. That is achieved by identifying the analgesic active components, integrating clinical trial data on their effectiveness, and relating known herb–drug interactions.

## 2. Materials and Methods

We carried out computerized literature searches on the PubMed electronic database. The herbs of interest were chosen based on herbal medicines currently available in the United States and commonly recommended by online search engines, which frequently influences how individuals chose their herbal treatment. The search keywords included “pain” combined with the herb of interest’s common name, Latin name, and common synonyms. All references published until 19, December, 2020 were included. Publications were initially screened by language, title, and abstract. Reference lists of articles identified from the initial search were also reviewed for further study. Clinical studies and systematic reviews on herbs used as a primary or a combined therapy compared to an active treatment or a placebo for all pain syndromes in humans fulfilled our inclusion criteria.

## 3. Results

### 3.1. St John’s Wort

**Figure 1:**
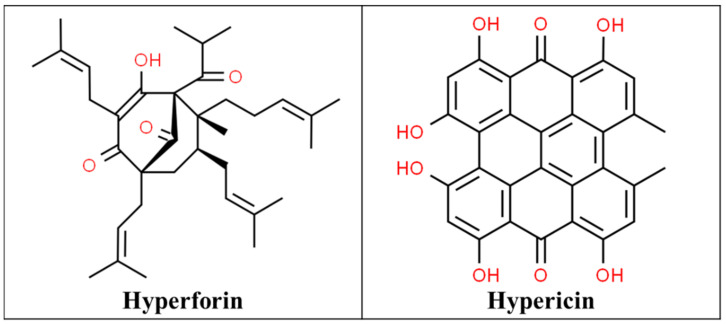
Chemical structures of hyperforin [12] and hypericin [13].

St John’s Wort (SJW) is extracted from the flowers and leaves of the plant *Hypericum perforatum* native to Asia and Europe, which was later introduced to North America by the Europeans. It includes at least 10 active constituents but the two principal pharmacological components are hyperforin and hypericin (Figure 1), which are responsible for their beneficial effects [9,10]. Hypericin can inhibit serotonin, norepinephrine, and dopamine reuptake, weakly inhibits monoamine oxidases (MAOIs) A and B and the crude extracts have a high affinity for gamma-aminobutyric acid (GABA) receptors. This has led to its role as an anxiolytic, sedative, antidepressant, and analgesic [11].

Among the herbs reviewed, SJW has the most potential for drug interaction. Adverse events are mostly due to its drug interaction with other selective serotonin reuptake inhibitors (SSRIs, such as paroxetine), MAOIs, opiates, tricyclic antidepressants, cold and flu medications which can lead to serotonin syndrome [14,15]. It has also been shown to be uterotonic in in vitro studies and rarely causes photosensitivity [10]. Its flavonoid components especially quercetin have been shown to have analgesic activity [16]. SJW has also been found to cause nausea, vomiting and anxiety when taken with sertraline, an antidepressant used in the treatment of chronic pain [17].

Hyperforin activates a regulator of the cytochrome P450 (CYP450) 3A4 transcription and results in the expression of 3A4 in the hepatocytes. St. John’s wort can also induce 2C9, 2D6, 2C19, 2E1, and 1A2. Concomitant use of opioids such as fentanyl, hydrocodone, codeine, tramadol, oxycodone and methadone with SJW may decrease the opioid concentrations and lead to withdrawal symptoms. Conversely, discontinuing the SJW may also cause increased opiate concentration causing toxicity [18,19,20,21]. The likely mechanism of this interaction between SJW and codeine is by inducing CYP3A4 metabolism, codeine conversion to the inactive norcodeine increases, resulting in less codeine being available for CYP2D6 to form its active metabolite morphine. It has also been reported that SJW active agents can inhibit MAOI A and B, but at concentrations up to 10 μM it may not be clinically relevant [22]. SJW’s analgesic effect plus its interaction with other analgesics such as fentanyl, morphine, ketamine, oxycodone has been studied in clinical trials.

Clinical studies and relevant review article are summarized in Table 1.

### 3.2. Ginger

**Figure 2:**
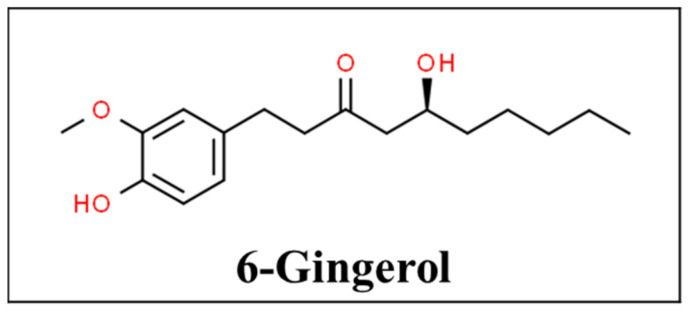
Chemical structure of 6-gingerol [39].

Ginger, also known as *Zingiber officinale*, has beautiful flowers but its tuberous rhizome has been used as a spice and medicine by herbalists mostly in India and China for the past 2500 years. The plant is found in most tropical countries [29,30]. It has uses for muscle pain and swelling, arthritis, headaches, digestive and appetite problems, prevention of motion sickness, postoperative nausea and vomiting, hyperemesis gravidarum, and also cold and bacterial infections due to its anti-oxidant mechanism [11,31]. Gingerols, especially 6-gingerol (Figure 2), are the active components of ginger. Ginger’s anti-emetic activity is not well understood but it is proposed to be caused by direct stimulation of the gastrointestinal tract or by antagonizing serotonin in the gut or central nervous system [32,33,34]. Its anti-inflammatory effects come from inhibiting arachidonic acid metabolism [33,34]. Based on the available data there is a delayed therapeutic action and therefore it does not help treat acute pain conditions such as exercise-induced muscle pain [35,36,37,38].

The adverse effects of ginger include drowsiness, excessive sedation, and arrhythmia [11], and perioperative physicians should be aware of its potent thromboxane-synthetase inhibition, which interferes with platelet aggregation and increases bleeding time based on in-vitro studies [40,41,42]. However, it has not been proven in in vivo human studies [43]. The concomitant use of ginger with other herbs or drugs with similar pharmacologic potential such as naproxen may increase the risk of bleeding and therapy modification should be considered [22]. If needed platelet functions status testing should be done before neuraxial or regional anesthetic procedures in any patient with bleeding or bruising history.

An animal study into ginger–drug interactions have found that combining acetaminophen with dried powdered ginger rhizome significantly enhanced acetaminophen’s anti-nociceptive effect and improved cognitive disturbances associated with chronic pain [44]. Another study noted that ginger root extract injected in rats before a sub-effective dose of morphine elicited a significant anti-nociceptive effect, higher than in groups treated with either morphine or the extract alone [45].

Clinical studies and relevant review articles are summarized in Table 2. (Table 2)

### 3.3. Turmeric

**Figure 3:**
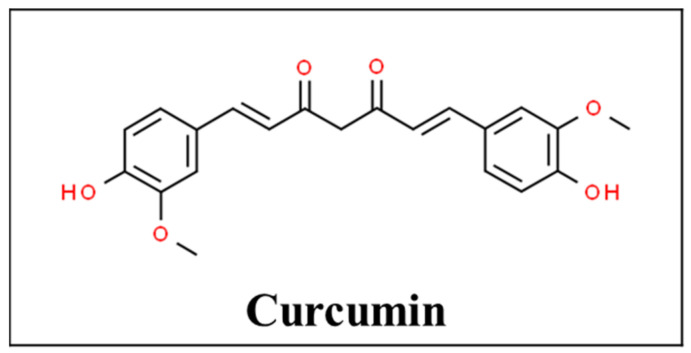
Chemical structure of curcumin [58].

The rhizome of the turmeric plant also known as *Curcuma longa* contains an active polyphenolic compound called curcumin (Figure 3). It has traditionally been used as an antiseptic, anti-inflammatory agent for wound healing as well as an antioxidant and analgesic agent [54,55]. Curcumin can regulate inflammatory cytokines such as interleukin (IL)-1 beta, IL-6, IL-12, Tumor necrosis factor (TNF)-alpha, interferon (IFN) gamma, and associated AP-1, NF-kappa B, and JAK-STAT signaling pathways. With its anti-inflammatory effects, it has been used in autoimmune diseases such as rheumatoid arthritis, inflammatory bowel disease, and multiple sclerosis [56]. In terms of drug interactions, turmeric supplementation of paclitaxel chemotherapy was found to improve the quality of life and pain scores in breast cancer patients [57].

Oral turmeric is well tolerated and safe for general use. Due to its poor bioavailability, higher doses are often used to achieve a systemic effect [59]. Studies have shown curcumin can inhibit platelet-activating factor and arachidonic acid platelet aggregation [60]. Due to its anti-thrombotic effects, concomitant use of turmeric with other drugs with similar pharmacologic potential such as naproxen may increase the risk of bleeding, and therapy modification is recommended.

Several pre-clinical studies have investigated interactions between curcumin and drugs. One such study in mouse models of acute nociceptive pain demonstrated a synergistic interaction in combination with pregabalin [61]. Curcumin was found to downregulate opioid-related nociceptin receptor 1 gene expression, which codes for nociceptin opioid peptide receptor (NOP), one of the four opioid receptors. This suggests an inhibitory effect on morphine-induced activation of the same gene, possibly decreasing tolerance and addiction to morphine and other analgesic opioids [62]. A synergistic anti-nociceptive effect was also noted in the combination of curcumin and diclofenac, an NSAID, in rats. Although curcumin did not produce significant alteration in oral diclofenac bioavailability, this interaction may have therapeutic advantages [63]. Another study on rats also noted that curcumin exhibited a synergistic interaction with a sub-analgesic dose of diclofenac [64].

Clinical studies and relevant review articles are summarized in Table 3. (Table 3)

### 3.4. Omega-3 Fatty Acids

**Figure 4:**
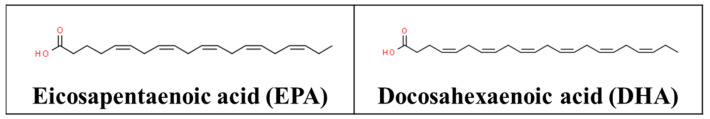
Chemical structures of eicosapentaenoic acid (EPA) [76] and docosahexaenoic acid (DHA) [77].

The effects of polyunsaturated fatty acids (PUFA) in reducing pain has been the focus of many studies. A dietary intake of n-3 series PUFA was demonstrated to help treat pain in conditions such as rheumatoid arthritis, neuropathy, dysmenorrhea, and inflammatory bowel disease. In patients with chronic pain, the levels of n-6 series polyunsaturated fatty acids are high, indicating a possible role in pain regulation [71]. One of the body’s natural omega-3 fatty acids (O3FA) is eicosapentaenoic acid (EPA, Figure 4) which can be found in various types of algae as well as in fish inhabiting cold deep waters. Studies have shown adding EPA to the diet can reduce the severity and frequency of migraine headaches likely by inhibiting prostaglandin levels and serotonin activity [72].

Based on studies and the prescribing information of O3FA, it may enhance the antiplatelet activity of drugs such as aspirin and naproxen and it is recommended to monitor the patients [73,74]. This is mediated by affecting platelet function and altering the interaction between platelets and the vascular wall [75].

Some animal studies have investigated the interaction between O3FA and drug products. For instance, the combination of O3FA and morphine in animal studies showed an additive anti-nociceptive effect, even showing analgesic activity at a sub-therapeutic dose of morphine. Moreover, chronic co-administration attenuated the development of tolerance to morphine [78]. Docosahexaenoic acid (DHA, Figure 4), a type of O3FA, has been studied in combination with drugs such as diclofenac. Animal studies on this combination attributed synergistic interaction at a systemic level in terms of nociception, inflammation and gastric security [79]. A similar study found that DHA combined with naproxen affords supra-additive nociception and gastric safety [80]. The same authors also noted synergistic anti-nociception and gastric safety with the combination of DHA with the NSAID indomethacin [81].

Clinical studies and relevant review articles are summarized in Table 4. (Table 4)

### 3.5. Capsaicin

**Figure 5:**
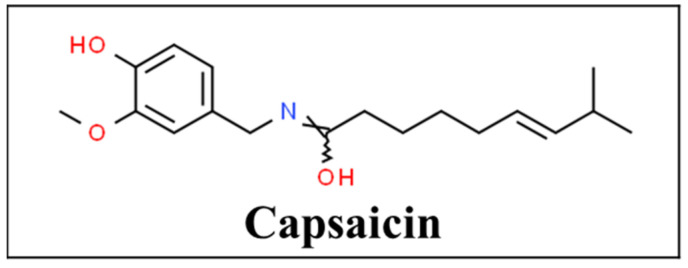
Chemical structure of capsaicin [97].

Capsaicin (Figure 5) is a natural chili pepper extract and its topical application is an established treatment option for various pain conditions [92]. Intense or repetitive exposure to capsaicin leads to a reversible and selective loss of nociceptive nerve endings. It specifically opens the nonselective cation ion, transient receptor potential cation channel subfamily V member 1 (TRPV1) also known as vanilloid receptor, predominantly found in C-fiber polymodal nociceptors. The influx of cations into the neurons leads to a transient burst of action potentials, leading to a profound burning, stinging, mechanical and thermal hyperalgesia. It is followed by reversible desensitization which can last for several weeks. This effect of capsaicin is referred to as “defunctionalization”. While pain receptors regenerate in 4 to 16 weeks, the pain fibers cannot transmit pain signals, resulting in a long-term decrease in sensitivity of mechanical, thermal, and noxious stimulation [93,94]. A randomized controlled trial found that supplementing topical diclofenac with capsaicin offered superior pain relief compared to diclofenac alone, but not compared to capsaicin alone [95]. In fact, topical capsaicin is well-tolerated in combination and no drug interactions have been noted so far [96].

Its cutaneous patches containing 8% capsaicin is approved in the European Union for the treatment of non-diabetic neuropathic pain [92,98].

Clinical studies and relevant review articles are summarized in Table 5. (Table 5)

### 3.6. Thunder God Vine

Thunder god vine is also known as *Tripterygium wilfordii* Hook F (TwHF) is a traditional Chinese herb. With its anti-inflammatory, immunosuppressive, and analgesic effects, it has been used for rheumatoid arthritis (RA) joint pain and insect pests [124,125]. It inhibits the expression of proinflammatory cytokines, proinflammatory mediators, adhesion molecules, and matrix metalloproteinases by lymphocytes, macrophages, synovial chondrocytes, and fibroblasts [126].

Oral TwHF has been associated with several adverse events including renal insufficiency, dysmenorrhea, decreased male fertility, hematotoxicity, embryotoxicity, and immune suppression demonstrated by an increased rate of infections. TwHF has a high risk-benefit ratio as its subacute toxicity demonstrated pathological changes in the reproductive and lymphatic systems [127]. It is recommended not to take TwHF with other immunosuppressive meds.

**Figure 6:**
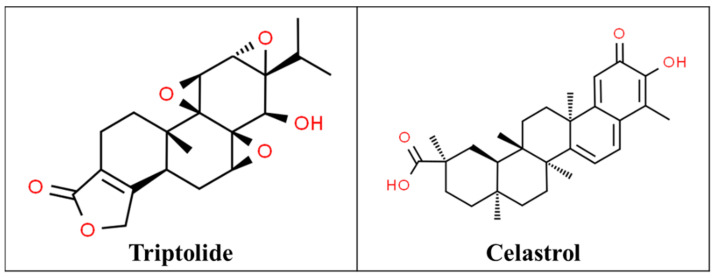
Chemical structures of triptolide [130] and celastrol [131].

Triptolide (Figure 6), an active component of TwHF, has demonstrated strong analgesic activity. Fluoxetine, an antidepressant used in the treatment of chronic pain, in combination with triptolide was shown to produce a significantly stronger analgesic effects than the use of fluoxetine alone, in an animal study [128]. A study done in the rat neuropathic pain model showed that the combination of triptolide and MK-801, a noncompetitive *N*-methyl-d-aspartate (NMDA) antagonist, produced synergistic analgesia, as well as inhibition of signaling pathways induced by chronic neuropathic pain [129].

It is worth noting that celastrol (Figure 6), another active component of TwHF, was found to signal through the cannabinoid receptor-2, a target of interest in drug development for pain relief [132].

Clinical studies and relevant review articles are summarized in Table 6. (Table 6)

### 3.7. Butterbur

**Figure 7:**
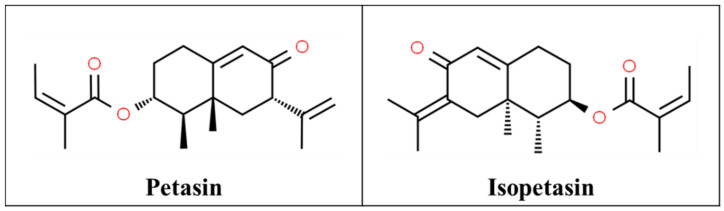
Chemical structures of petasin [136] and isopetasin [137].

Butterbur is the root extract of *Petasites hybridus*, a perennial shrub that was used since ancient times for its medicinal properties such as fever, wound healing, muscle spasm, and migraine prophylaxis. The active agents are likely its sesquiterpenes such as petasin and isopetasin (Figure 7) [72,134]. Isopetasin can activate transient receptor potential ankyrin 1 (TRPA1) channels, resulting in neuropeptide containing nociceptor excitation and consequently heterologous neuronal desensitization. Petasites may also act through calcium channel regulation and peptide-leukotriene biosynthesis inhibition. These effects on pain and neurogenic inflammation may count for its role as an anti-migraine treatment [72,135].

Its preparation is very important considering its leaves contain a high concentration of pyrrolizidine alkaloids, which are hepatotoxic and carcinogenic. The most common adverse events of butterbur are mild gastrointestinal symptoms and belching [134].

Petadolex, a butterbur extract with clinically proven efficacy against migraines has been associated with cases of herbal-induced liver injury. Among the most severe incidences, 50% of patients were co-medicating with NSAIDs or zolmitriptan, which may have contributed or even elicited liver injury. [138]. The potential hepatotoxicity of butterbur has been associated with the pyrrolizidine alkaloids, which were removed from many commercially available presentations [139,140,141]. Pyrrolizidine alkaloids are metabolized to toxic metabolites by CYP3A4, so concomitant administration of CYP3A4 inducers may enhance the toxicity of butterbur [142].

Special extracts of the rhizomes of butterbur were found to inhibit of prostaglandins E_2_ and COX-2 release by direct interaction with the enzymes [143]. This suggests an additive effect when combined with certain NSAIDs.

Clinical studies are summarized in Table 7. (Table 7)

### 3.8. Feverfew

**Figure 8:**
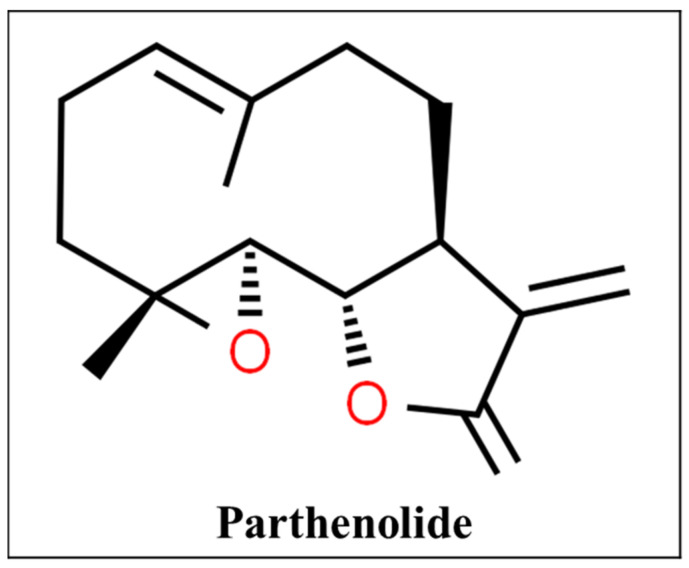
Chemical structure of parthenolide [148].

Feverfew constitutes the dried leaves of the weed plant *Tanacetum parthenium*. Several centuries ago it was used to treat fever, headaches, and inflammation. It was rediscovered in the late 20th century for migraine headaches. Its active agents are the parthenolide (Figure 8) within the leaves. It can inhibit serotonin release from white blood cells and platelets, and prevent platelet aggregation. It can also have anti-inflammatory action by inhibiting phospholipase A and prostaglandin synthesis [72].

Adverse events documented in clinical trials include mouth ulcers, gastrointestinal disturbances, and joint aches [72]. By inhibiting platelet aggregation and secretion it can increase the risk of bleeding when used concomitantly with other antiplatelet or anticoagulant agents such as naproxen. Therefore, therapy modification is recommended [22].

Feverfew was found to strongly inhibit the activity of CYP3A4, the most abundant cytochrome found in livers and small intestines, responsible for the metabolism of more than 50% of the currently used therapeutic drugs [149].

Clinical studies and relevant review articles are summarized in Table 8.

### 3.9. Willow Bark

**Figure 9:**
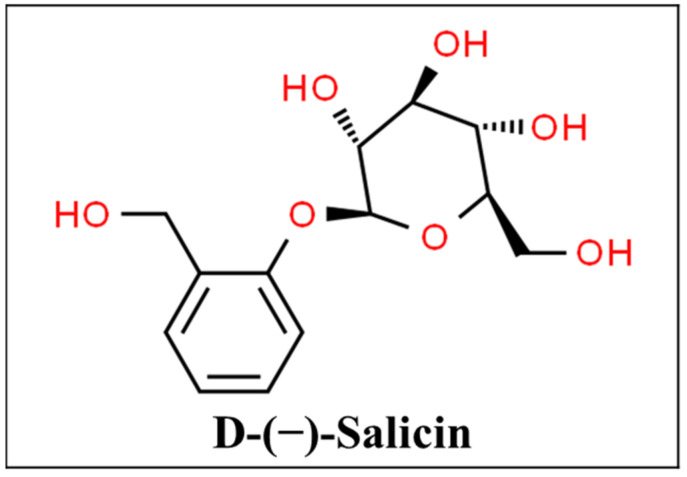
Chemical structure of salicin [158].

Willow bark extract is one of the first examples of modern medication development from an herbal drug. It is obtained from the willow tree also known as *Salix*, and is generally standardized to salicin (Figure 9) but may contain other salicylates as well as flavonoids and polyphenols. It has been used for thousands of years for its antipyretic, analgesic, and anti-inflammatory effects [155,156]. The active agents of willow bark extract inhibit COX-2 mediated release of prostaglandins E_2_ and the release of interleukin 1ß and tumor necrosis factor-α [157].

The most common reported adverse events are gastrointestinal disturbances, allergic reaction to salicylates, and children are at risk of Reye’s syndrome. It should be avoided in pregnant females as salicylates can cross the placenta and are eliminated slowly in newborns. There is an increased risk of bleeding and concomitant treatment with other salicylate-containing medication increases these risks and therapy should be monitored or modified [155,159]. NSAIDs also have the potential to interact with herbal supplements that are known to possess antiplatelet activity, such as willow bark [160]. Nonetheless, no relevant drug interactions were reported in a trial on the aqueous willow bark extract STW 33-I, in patients allowed to co-medicate with NSAIDs and opioids [161]. The incidences of hepatotoxicity and nephrotoxicity may be augmented by acetaminophen when concomitantly used with herbs containing salicylate, such as willow bark [160].

Clinical studies and relevant review articles are summarized in Table 9. (Table 9)

## 4. Discussion

Pain is one of the most common conditions for which patients in the United States turn to CAM. In turn, one of the most commonly sought forms of CAM is herbal medicine. These therapeutic avenues are often underreported or undisclosed to medical professionals, which may cause risks to the patients. CAM are not only largely unregulated, but their efficacy and safety are often poorly understood. Herbal medicines in particular often contain combinations of active components, and their sourcing presents possible risks related to contamination with toxins. Additional risks come from combination therapy, as some herbal medicines have been shown to affect the safety and efficacy of conventional medicines found over-the-counter or prescribed by physicians. Our review aims to consolidate efficacy and safety data for some of the most commonly used herbal medicines for pain management; by identifying the active components to which are attributed analgesic effects, integrating clinical trial data on their effectiveness, and relating known herb–drug interactions.

Herbal medicines are often used with little physician knowledge or guidance. A better understanding of the active ingredients and mechanisms of action of common herbal medicines can guide practitioners to modify their treatment plans, determine appropriate use, anticipate toxicities, and prevent possible adverse herb–drug interactions for patients. Chronic pain can become debilitating, and patients are often motivated to seek alternative treatment, including herbal medicine. While self-management has been recognized as part of a successful overall treatment strategy for patients suffering from chronic pain [3], it is important for physicians to be able to optimize and integrate herbal medicines and, if needed, other CAM into the care they provide.

The largely unregulated status of herbal medicines may present safety issues. In particular, herbal medicines are often taken alongside synthetic drugs, enabling herb–drug interactions with poorly studied clinical outcomes. Concurrent use of agents with similar pharmacological effects such as anti-inflammatory, hepatotoxic, anti-platelet, or anticoagulation should be approached with caution and if needed the patient should be monitored or the treatment plan should be modified. In fact, drug classes most likely to interact with herbs are antiplatelets, anticoagulants, sedatives, antidepressants, and antidiabetics [22]. Concurrent use of ginger, turmeric, omega-3 fatty acid, feverfew, and willow bark with agents that predispose to bleeding, can enhance their effect and increase the risk of bleeding. Therefore, the patients should be monitored and if needed the treatment plan should be modified. Other commonly noted interactions are due to herbal medicines modulating the expression and activity of cytochromes. Nonetheless, there are also instances of additive or synergistic outcomes from herb–drug interactions.

With the popularity of herbal medicine growing annually, our review provides a summary and an overview of available data on the common herbs of interest used as alternatives for pain management. However, further rigorous scientific and systematic inquiries are necessary to be able to validate or refute the clinical claims made for herbal medicine.

## Figures and Tables

**Table 1:** Clinical studies and review article on St John’s Wort and pain management.

Author	Journal and Year of Publication	Type of Study	Number of Patients, Duration of Study	Study Groups, Active Agent and Placebo Dosage	Key Finding(s)
**Clinical Studies**					
Loughren, M.J. et al.	*Anesthesiology* 2020	Randomized parallel-group clinical trial	16 human volunteers, 35 days study, 21 days of treatment, 12 h follow up after intravenous fentanyl administration	Healthy volunteers receiving SJW tablet 300 mg thrice daily (*n* = 8) versus placebo tablet (*n* = 8)	SJW does not alter fentanyl pharmacodynamics or clinical effects, likely does not affect hepatic clearance or blood-brain barrier efflux. Taking SJW will likely not respond differently to intravenous fentanyl for analgesia or anesthesia [23].
Galeotti, N. et al.	*Journal of Pharmacological Sciences* 2014	Clinical trial with one-way analysis of variance (ANOVA) for repeated measures	8 human volunteers, 170 rodents, 5 days treatment, up to 1 h follow up	Rodents (adult male Swiss albino mice and rats), healthy male volunteers receiving oral morphine co-administered with SJW 300 mg tablets or morphine alone	A low dose of SJW significantly potentiated oral 10 mg morphine’s analgesic effect in humans and rodents [24].
Peltoniemi, M. A. et al.	*Fundamental and Clinical Pharmacology* 2012	Placebo-controlled, randomized, cross-over clinical trial	12 healthy nonsmoking volunteers, 14 days SJW phase and 14 days placebo phase with 4 weeks interval, 24 h follow up	Oral SJW 300 mg versus placebo, six women and six men	SJW decreased the exposure to oral S-ketamine but was not associated with significant changes in the analgesic or behavioral effects of ketamine. Usual doses of S-ketamine if used concomitantly with SJW may become ineffective [25].
Clewell, A. et al.	*Journal of Drugs in Dermatology* 2012	Prospective, randomized, multi-centered, comparative, open-label clinical study	149 (120 participants received treatment), 14 days treatment and follow up	Patients with active HSV-1 and HSV-2 lesions receiving single use of Dynamiclear formulation (*n* = 61), and patients receiving repeat topical 5% Acyclovir (*n* = 59)	A single application of topical formulation containing *hypericum* *perforatum* and copper sulfate is helpful in the management of burning and stinging sensation, erythema, vesiculation, and acute pain caused by HSV-1 and HSV-2 lesions in adults [26].
Nieminen, T. H. et al.	*European Journal of Pain* (London, England) 2010	Placebo-controlled, randomized, cross-over clinical trial	12 healthy, non-smoking volunteers, 15 days SJW phase and 15 days control phase with 4 weeks interval, up to 48 h follow up	6 women and 6 men, oral SJW 300 mg versus placebo thrice daily	SJW significantly decreased the plasma concentrations of oral oxycodone 15 mg and also the self-reported drug effect. This may be of clinical significance in chronic pain management [21].
Sindrup, S. H. Et al.	*Pain* 2001	Randomized, double-blind, placebo-controlled and cross-over	54 (47 patients completed the study), 2 treatment period of 5 weeks duration	Oral SJW (2700 µg total hypericin daily, *n* = 27)) versus placebo tablet (*n* = 27), up to 6 paracetamol 500 mg tablets could be used daily as escape medication	There was a trend of lower total pain score with oral SJW compared to the placebo group. However, none of the volunteers’ pain ratings were significantly changed by SJW as compared to placebo. Complete, good, or moderate pain relief was reported by nine patients taking SJW and two with placebo. In conclusion, SJW does not have a significant effect on pain due to polyneuropathy [27].
**Review Article**					
Galeotti, N.	*Journal of Ethnopharmacology* 2017	Review Article	-	Preclinical animal, in vivo and in vitro studies, clinical trials with dental pain	Preclinical animal studies showed the potential of low doses SJW dry extracts to induce antinociception, mitigate acute and chronic hyperalgesic states, and to augment opioid analgesia. Clinical studies showed SJW is promising in dental pain conditions. SJW analgesia appears at low doses (5–100 mg/kg), decreasing the risk of herbal–drug interactions caused by hyperforin, a potent CYP enzymes inducer [28].

**Table 2:** Clinical studies and review articles on ginger and pain management.

Author	Journal and Year of Publication	Type of Study	Number of Patients, Duration of Study	Study Groups, Active Agent and Placebo Dosage	Key Finding(s)
**Clinical Studies**					
Martins, L. B. et al.	*Cephalalgia: an International Journal of Headache* 2018	Double-blind placebo-controlled randomized clinical trial	60 patients, 2 h session, up to 48 h follow-up	Emergency room patients suffering from migraine headaches received 400 mg of ginger extract capsules (5% active gingerols or 20 mg, *n* = 30) or placebo (cellulose, *n* = 30), in addition to an intravenous ketoprofen 100 mg	Patients treated with ginger and NSAIDs for migraine attacks had significantly better clinical responses [46].
Rondanelli, M. et al.	*Natural Product Research* 2017	Prospective trial	15 patients, 30 days treatment and follow up	13 women and 2 men with chronic inflammation, pain due to knee arthrosis and nonsteroidal anti-inflammatory drugs NSAIDs poor responders receiving highly standardized ginger 25 mg and Echinacea 5 mg extract supplementation	A decrease in 0.52 cm in knee circumference (*p* < 0.01), SF-36 (*p* < 0.05), and a significant improvement of 12.27 points was observed for Lysholm scale score (*p* < 0.05) [47].
Black, C. D. et al.	*The Journal of Pain: Official Journal of the American Pain Society* 2010	Double-blind, placebo controlled, randomized experiments	Study 1: 34 patients Study 2: 40 patients, 11 consecutive days for each study	Study 1: 2 g of Raw ginger (*n* = 17) versus placebo (*n* = 17) Study 2: 2 g of heat-treated ginger supplementation (*n* = 20) versus placebo (*n* = 20) on muscle pain	Patients taking daily raw and heat-treated ginger had a moderate-to-large reduction in muscle pain following exercise-induced muscle injury. Ginger has hypoalgesic effects in patients with osteoarthritis and is an effective pain reliever [37].
Black, C. D. et al.	*Phytotherapy Research: PTR* 2010	Double-blind, cross-over design	27 patients, 3 consecutive days	13 men and 15 women receiving 2 g of ginger or 2 g of placebo (white flour) 24 h and 48 h after exercise	Ingestion of a single 2 g dose of ginger does not lessen inflammation, dysfunction, and eccentric exercise-induced muscle pain. However, ginger may decrease day-to-day muscle pain progression [36].
Black, C. D. et al.	*International Journal of Sport Nutrition and Exercise Metabolism* 2008	Double-blind, crossover design	25 patients, 1 day preliminary testing and 2 days of experimental testing	10 men and 15 women receiving 2 g dose of oral ginger or 2 g placebo (flour)	Consumption of ginger had no clinically or statistically significant effect on perceptions of muscle pain during exercise compared with placebo [35].
Cady, R. K. et al.	*Medical Science Monitor: International Medical Journal of Experimental and Clinical Research* 2005	Open-label study	29 patients, one session with 24 h follow up	24 females and 5 males with a history of migraines receiving two 2 mL sublingual doses of GelStat Migraine (3× feverfew, 2× ginger) 5 min apart, second treatment could be used between 60 min and 24 hr, subjects were also allowed to take usual migraine therapy 2 hr after initial dose of study medication	Administration of sublingual ginger and feverfew compound (GelStat Migraine) early during the mild headache phase is effective as a first-line abortive treatment for acute migraine [48].
Altman, R. D. et al.	*Arthritis and Rheumatism* 2001	Randomized, double-blind, placebo-controlled, multicenter, parallel-group, 6-week study trial	261 (247 patients completed the study), 6 weeks of treatment and follow up	Patients receiving ginger extract (255 mg of EV.EXT 77, *n* = 130) versus patients receiving placebo capsules (coconut oil, *n* = 131) twice daily, Acetaminophen was permitted as rescue medication	The highly purified and standardized ginger extract had a statistically significant moderate effect on reducing symptoms of knee osteoarthritis with a good safety profile (mostly mild gastrointestinal adverse events in the ginger extract group) [49].
Bliddal, H. et al.	*Osteoarthritis and Cartilage* 2000	Randomized, controlled, double blind, double dummy, cross-over study	75 (56 patients completed the study), 3 treatment periods of 3 weeks each.	15 men and 41 women with osteoarthritis of the hip or knee receiving ginger extract (170 mg EV.ext-33) versus placebo versus ibuprofen 400 mg thrice daily, Acetaminophen maximum 3 gr daily as rescue drug	In the first period of treatment before cross-over, a statistically significant effect of the ginger extract was demonstrated by explorative statistical methods. However, a significant difference was not observed in the study as a whole [50].
**Review Articles**					
Chen, C. X. et al.	*Evidence-Based Complementary and Alternative Medicine: eCAM* 2016	Systematic review and meta-analysis of 6 randomized controlled trials (RCTs)	598	Women with dysmenorrhea taking oral ginger (750–2500 mg daily) versus placebo or active treatment (ibuprofen, NSAIDs, mefenamic acid, zinc, or progressive muscle relaxation)	Oral ginger can be an effective treatment for menstrual pain in dysmenorrhea. However, due to the small number of studies, high heterogeneity across trials, and poor methodological quality of the studies the findings need to be interpreted with caution [51].
Wilson, P. B.	*Journal of Strength and Conditioning Research* 2015	Systematic review of randomized trials (6 analgesic and 8 ergogenic articles)	Analgesic: 175 Ergogenic Aid: 106	Randomized crossovers or parallel receiving ginger (2–4 gr daily) or placebo	Taking ginger over 1–2 weeks may reduce pain from prolonged running and eccentric resistance exercise. However, its safety and efficacy as an analgesic for a wide range of athletic endeavors need to be further studied [52].
Terry, R. et al.	*Pain Medicine* (Malden, Mass.) 2011	Systematic review (7 articles)	481	Human adults suffering from any pain condition, taking oral *Z. officinale*, treatment against a comparison condition	Seven published articles, reporting eight trials were included in the review and assessed using the Jaded scale. The use of *Z. officinale* for the treatment of pain cannot be recommended at present due to a paucity of well-conducted trials [38].
Leach, M. J. et al.	*JBI Library of Systematic Reviews* 2008	Systemic review of 3 randomized clinical trials	680	Patients with osteoarthritis receiving ginger versus placebo versus ibuprofen	The evidence for administration of ginger in adults with osteoarthritis of the knee and/or hip is weak, due to significant heterogeneity between the studies. It is recommended to improve the research design, instrumentation, and ginger dosage, which may help to demonstrate the safe and effective use of ginger in patients with osteoarthritis [53].

**Table 3:** Clinical studies and review articles on turmeric and pain management.

Author	Journal and Year of Publication	Type of Study	Number of Patients, Duration of Study	Study Groups, Active Agent and Placebo Dosage	Key Finding(s)
**Clinical Studies**					
Haroyan, A. et al.	*BMC Complementary and Alternative Medicine* 2018	Three-arm, parallel-group, randomized, double-blinded, placebo-controlled trial	201 patients, 12 weeks treatment and follow up	Patients receiving Curamin 500 mg capsules (*n* = 67) versus Curamed 500 mg capsules (curamin combined with boswellic acid, *n* = 66) versus placebo capsules (500 mg excipients, *n* = 68) thrice daily	Administration of curcumin complex or its combination with boswellic acid for twelve-weeks reduces pain-related symptoms in patients with osteoarthritis compared to placebo. Curcumin in combination with boswellic acid is more effective presumably due to synergistic effect [65].
Appelboom, T. et al.	*The Open Rheumatology Journal* 2014	Retrospective observational study	820 patients, 6 months of treatment and up to 6 months follow up after treatment	Patients with joint problems treated with the curcumin preparation Flexofytol^®^ (42 mg curcumin, 739 were using 2 capsules two times daily and 81 took 2 capsules 3 times daily)	Flexofytol^®^ is a potential nutraceutical for patients with joint problems, with great tolerance and rapid effect for pain, articular mobility, and quality of life [66].
Kalluru, H. et al.	*Complementary Therapies in Clinical Practice* 2020	Prospective, consecutive case series	60 patients, 21 days treatment	Breast cancer patients receiving combination of turmeric supplementation 2 g daily and paclitaxel chemotherapy	Turmeric supplementation improved quality of life scores and led to clinically relevant and statistically significant improvement in global health status, symptom scores (fatigue, nausea, vomiting, pain, appetite loss, insomnia), and hematological parameters [57].
**Review Articles**					
Eke-Okoro, U. J. et al.	*Journal of Clinical Pharmacy and Therapeutics* 2018	Systemic review of pre-clinical and clinical studies	921	Observational or turmeric versus placebo or active controls	Turmeric (curcumin) may be used as a sole analgesic or as a combination of opioid, NSAIDs, or paracetamol (acetaminophen) sparing strategies [67].
Gaffey, A. et al.	*JBI Database of Systematic Reviews and Implementation Reports* 2017	Thirteen studies both experimental and epidemiological study designs including RCTs, non-RCTs, quasi-experimental and before and after studies	1101	Patients with musculoskeletal pain receiving curcuminoids versus placebo versus NSAIDs versus curcuminoid-containing herbomineral mixtures versus placebo or active controls	In musculoskeletal pain conditions, there is insufficient evidence to recommend administration of curcuminoids to relieve pain and improve function. This is due to limitations caused by the small number of relevant studies, small sample sizes, variability in study quality, short interventional durations, gender-bias toward females, and lack of long-term data extraction [68].
Perkins, K. et al.	*Journal of Evidence-Based Complementary & Alternative Medicine* 2017	Review of 8 clinical trials	874	Observational or turmeric versus placebo or active controls	Published clinical trials demonstrate similar efficacy of *Curcuma* formulations compared to NSAIDs and potentially to glucosamine in treating osteoporosis symptoms. While there was statistical significance in the majority of the studies, due to major study limitations and the small magnitude of the effect, the validity of the results are questionable and further rigorous studies are needed before recommending *Curcuma* as an effective therapy for knee osteoarthritis [69].
Daily, J. W. et al.	*Journal of Medicinal Food* 2016	Systematic review and meta-analysis of randomized clinical trials	1874	Patients receiving turmeric extract versus placebo or active controls	The studies provide evidence for the efficacy of about 1000 mg/day of curcumin in treating arthritis. However, due to the insufficient total number of RCTs in this analysis, the methodological quality of the primary studies, and total sample size, a definitive conclusion can not be made. Further rigorous and larger studies are required to confirm the efficacy of turmeric in treating arthritis [70].

**Table 4:** Clinical studies and review articles on omega-3 fatty acid and pain management.

Author	Journal and Year of Publication	Type of Study	Number of Patients, Duration of Study	Study Groups, Active Agent and Placebo Dosage	Key Finding(s)
**Clinical Studies**					
Ruiz-Tovar, J. et al.	*Clinical Nutrition* (Edinburgh, Scotland) 2018	Prospective randomized clinical trial	40 patients, treatment was started 10 days up to 8 h before surgery, 30 days follow up after surgery	Patients receiving a preoperative balanced energy high-protein (10 g/100 mL) formula (control group, *n* = 20) and patients receiving the same preoperative nutritional formula enriched with O3FA (2 g EPA/400 mL) (experimental group, *n* = 20)	A preoperative diet enriched with O3FA is associated with reduced postoperative pain, decreased postoperative levels of C-reactive protein, and a greater preoperative weight loss in patients undergoing Roux-en-Y gastric bypass [82].
Lustberg, M. B. et al.	*Breast Cancer Research and Treatment* 2018	Randomized, double-blind, placebo-controlled	44 (35 women completed the study), 24 weeks treatment and follow up	Comparing 4.3 g/day of n-3 PUFA supplements (*n* = 22) versus placebo (mixture of fats and oils, *n* = 22)	High-dose n-3 PUFA supplementation taken concomitantly with aromatase inhibitors (AIs) is well tolerated and feasible in postmenopausal breast cancer. However, the mean pain severity scores using the Brief Pain Inventory-Short Form (BPI-SF) for joint symptoms did not change significantly by time or treatment arm. Further studies are needed to evaluate its efficacy in preventing joint symptoms [83].
Hershman, D. L. et al.	*Journal of Clinical Oncology: Official Journal of the American Society of Clinical Oncology* 2015	Randomized multicenter placebo-controlled trial	249 patients, 24 weeks treatment and follow up	3.3 g/day O3FA (*n* = 122) versus placebo (blend of soybean and corn oil, *n* = 127)	There were a considerable (>50%) and sustained improvement in aromatase inhibitor arthralgia for both O3FAs and placebo but based on BPI-SF scores there was no meaningful difference between the groups [84].
Ko, G. D. et al.	*The Clinical Journal of Pain* 2010	Clinical series	5 patients, up to 19 months of treatment and follow up	Five patients receiving high oral doses of omega-3 fish oil (2400–7200 mg/day)	There was improved function and clinically significant pain reduction up to 19 months after treatment initiation. Therefore, O3FAs may be beneficial in treating neuropathic pain in patients with cervical radiculopathy, carpal tunnel syndrome, thoracic outlet syndrome, fibromyalgia, and burn injury. Further studies with RCTs in a more specific neuropathic pain population are required [85].
Maroon, J. C. et al.	*Surgical Neurology* 2006	Prospective study	250 patients, average of 75 days treatment, one month follow up	Patients who had nonsurgical neck or back pain receiving a total of 1200 mg/day of omega-3 EFAs	Taking O3FA supplement demonstrated equivalent effect in reducing arthritic pain compared to NSAIDs. O3FA fish oil supplements are a safer alternative to NSAIDs in treating nonsurgical neck or back [86].
Tomer, A. et al.	*Thrombosis and Haemostasis* 2001	Double-blind, controlled clinical trial	20 patients, 1 year treatment and follow up	O3FA (0.1 g/kg/day, *n* = 5) versus olive oil (0.25 g/kg/day, *n* = 5) versus asymptomatic control group without sickle cell disease (*n* = 10)	Dietary n-3FAs reduce the frequency of pain episodes in sickle cell disease likely by reducing prothrombotic activity [87].
**Review Articles**					
Wojcikowski, K. et al.	*Alternative Therapies in Health and Medicine* 2018	Review of literature	-	Patients with chronic pain	There are several complementary therapies that may be effective in reducing chronic pain or the need for analgesics. Some of these therapies include O3FAs, curcumin, and capsaicin. Multimodal individualized treatment for each patient is recommended [88].
Abdulrazaq, M. et al.	*Nutrition* (Burbank, Los Angeles County, Calif.) 2017	Review article of 18 RCTs	1143	Patients receiving omega-3 PUFAs versus placebo or active controls	Omega-3 PUFAs may decrease pain associated with rheumatoid arthritis. Doses of 3 to 6 g/day have a greater effect. However, Due to the limitations found in the reviewed RCTs including small populations and short study periods, further research is needed [89].
Prego-Dominguez, J. et al.	*Pain Physician* 2016	Meta-analysis, systematic review	985	Patients receiving O3FA or olive oil	Omega-3 PUFA supplementation can moderately improve chronic pain, mostly due to dysmenorrhea. More studies on the preventive potential of PUFA supplementation is needed [90].
Goldberg, R. J. et al.	*Pain* 2007	Meta-analysis of 17 RCTs	823	Patients receiving Omega-3 PUFA or olive oil	Omega-3 PUFA supplementation for at least 3 months demonstrated improvement in joint pain caused by rheumatoid arthritis, or joint pain secondary to inflammatory bowel disease, and dysmenorrhea [91].

**Table 5:** Clinical studies and review articles on capsaicin and pain management.

Author	Journal and Year of Publication	Type of Study	Number of Patients, Duration of Study	Study Groups, Active Agent and Placebo Dosage	Key Finding(s)
**Clinical Studies**					
Galvez, R. et al.	*The Clinical Journal of Pain* 2017	Prospective, open-label, observational study	306 patients, repeat treatments and follow up over 52 weeks	Patients with posttraumatic or postsurgical nerve injury, postherpetic neuralgia, human immunodeficiency virus (HIV) associated distal sensory polyneuropathy, or other peripheral neuropathic pain receiving </= 6 capsaicin 8% patch with retreatment at 9 to 12 week intervals	Capsaicin 8% patch is well-tolerated with a variable alteration in sensory function and nominal risk for complete sensory loss [99].
Mankowski, C. et al.	*BMC Neurology* 2017	Cohort, open-label, observational, multicenter, European non-interventional study	429 (420 patients received at least one treatment), </= 52 weeks treatment and followup	Patients with non-diabetes-related peripheral neuropathy (PNP) received up to 4 capsaicin 8% patch (179 mg of capsaicin) per treatment (at least 90 days interval)	Capsaicin 8% patch is effective, generally well-tolerated, and can result in sustained pain relief, significant improvement in overall health status and quality of life [100].
Zis, P. et al.	*Pain Physician* 2016	Prospective open-label study	90 patients, single treatment and follow up at 2, 8, and 12 weeks	Patients with lumbosacral pain received capsaicin 8% patch	Treatment with capsaicin 8% patch resulted in substantial neuropathic pain relief and improved quality of life. The results should be further evaluated in a prospective randomized placebo-controlled study [101].
Campbell, C. M. et al.	*Pain* 2016	Randomized double-blind placebo-controlled study	58 patients, single treatment, 4 weeks follow up	A single capsaicin 0.1 mg dose (*n* = 30) versus placebo (*n* = 28) injected into the region of the Morton’s neuroma	A trend toward significance was found in the second and third week. In the capsaicin-treated group, there was a reduction in oral analgesics and improvement in functional interference scores. Based on the findings, injection of capsaicin in painful intermetatarsal neuroma is an effective treatment [102].
Haanpaa, M. et al.	*European Journal of Pain* (London, England) 2016	Open-label, randomized, multicenter, non-inferiority trial.	629 (559 patients received study medication), 8 weeks follow up	Patients received either the capsaicin 8% patch (1 to 4 patches, *n* = 282) or an optimized dose of oral pregabalin (75 mg/day up to 600 mg/day, *n* = 277)	Capsaicin 8% patch resulted in a faster onset of action, fewer systematic adverse events, greater treatment satisfaction, and non-inferior pain relief compared to an optimized dose of pregabalin in patients with PNP [103].
Raber, J. M. et al.	*Acta Neurologica Belgica* 2015	Clinical trial	37 patients received single treatment, observed 4 weeks prior to 12 weeks post administration	Patients suffering from painful, distal symmetric polyneuropathy for an average of 5 years. Single application of the capsaicin 8% cutaneous patch (Qutenza™)	The capsaicin 8% cutaneous patch resulted in a substantial relief of neuropathic pain, a prolongation of sleep mainly in patients with HIV infection, decreased oral pain medication consumption, and a resumption of social activities [104].
Maihofner, C. G. et al.	*European Journal of Pain* (London, England) 2014	Prospective multicenter RCT	1063 (1044 patients evaluated for effectiveness), single application, 12 weeks follow up	Non-diabetic patients with peripheral neuropathic pain. single application of capsaicin 8% cutaneous patch	The highest treatment response was observed with the capsaicin 8% cutaneous patch in patients suffering from peripheral neuropathic pain of fewer than 6 months. This shows early initiation of topical treatment is recommended [105].
Bischoff, J. M. et al.	*PLoS One* 2014	Randomized, double-blind, placebo-controlled trial	46 patients, 3 months follow up	Patients suffering from inguinal post-herniorrhaphy pain received either an inactive placebo patch (*n* = 22) or a capsaicin 8% patch (*n* = 24)	A trend toward pain improvement was observed in patients treated with capsaicin 8% patch after 1 month, but there were no significant differences in pain relief between the capsaicin and placebo group [106].
Hoper, J. et al.	*Current Medical Research and Opinion* 2014	Prospective non-interventional trial	1044 patients (822 patients completed the pain-DETECT questionaire at baseline, 571 completed questionaire at baseline and week 12), 12 weeks follow-up	Patients with peripheral neuropathic pain treated with capsaicin 8% cutaneous patch. Single application of up to 4 patches, applied 30 min for feet or 60 min other body parts.	Applying topical capsaicin 8% in patients with peripheral neuropathic pain effectively reduced sensory abnormalities. Completion of the pain-DETECT questionnaire was optional and therefore the data was incomplete and not available for all patients. Further studies are needed to confirm these results [107].
Maihofner, C. et al.	*Current Medical Research and Opinion* 2013	Prospective, non-interventional study	1040 patients, single application, 12 weeks follow up	Patients with peripheral neuropathic pain received single capsaicin 8% patch application of up to 4 patches	Application of capsaicin 8% patch is safe and effective. Because there was no control group, a comparison of the study results with that of therapeutic alternatives is not justified [108].
Irving, G. et al.	*The Clinical Journal of Pain* 2012	Double blind, randomized controlled studies	1127 patients, single 60 min treatment, 12 weeks follow up	Patients suffering from postherpetic neuralgia on at least 1 systemic neuropathic pain medication: 302 patients received capsaicin 8% patch and 250 control (capsaicin, 0.04%). Patients not on systemic neuropathic pain medication: 295 received capsaicin 8% patch and 280 control	A single capsaicin 8% patch for 60-min reduces postherpetic neuralgia for up to 12-weeks regardless of concomitant use of systemic neuropathic pain medication [109].
Webster, L. R. et al.	*Diabetes Research and Clinical Practice* 2011	Open-label, randomized, uncontrolled clinical trial	117 patients, 12 weeks follow up	Patients were pre-treated with one of three 4% lidocaine topical anesthetics (Topicaine Gel, or Betacaine Enhanced Gel 4, or L.M.X.4,) followed by single capsaicin 8% patch application for 60- or 90- min	Applying capsaicin 8% patch with any of the three topical anesthetics was well-tolerated, generally safe, and reduced pain over 12 weeks in patients with postherpetic neuralgia and painful diabetic neuropathy [110].
Hartrick, C. T. et al.	*Clinical Drug Investigation* 2011	Randomized, placebo-controlled, parallel-group, double-blind study	14 patients, 42 days follow up	Patients received direct instillation of capsaicin 15 mg (Anesiva 4975, *n* = 7) or placebo (*n* = 7) into the surgical site immediately prior to total knee arthroplasty wound closure, postoperative IV morphine for 24 hr and oral oxycodone as rescue medication were provided	While patients receiving capsaicin had higher BMIs, they had comparable or better pain scores, longer-lasting effect, improved active range of motion at 14 days, less opioid use in the first 3 postoperative days, and less pruritis which likely is related to the opioid-sparing effect [111].
Chrubasik, S. et al.	*Phytotherapy Research: PTR* 2010	Randomized double-blind multicenter study	281 patients treated (only 130 included in analysis), 3 weeks of treatment and follow up	Patients receiving capsaicin 0.05% cream (Finalgon ^®^ CPDWarmecreme, *n* = 140) or placebo (*n* = 141)	Capsaicin cream is effective and well-tolerated in patients with chronic soft tissue pain and patients with chronic back pain compared to the placebo group [112].
Cianchetti, C	*International Journal of Clinical Practice* 2010	Single-blinded placebo-controlled cross-over study	23 patients, 30 min follow up after application of treatment	20 females and 3 males with painful arteries in absence of migraine attack receiving topical capsaicin 0.1% or vaseline jelly	While the number of patients was small, results show topical capsaicin is effective in relieving arterial pain during and in the absence of migraine attacks [113].
Aasvang, E. K. et al.	*Anesthesia and Analgesia* 2008	Single-center, randomized, double-blind, placebo-controlled study	41 patients, Single treatment, 4 weeks follow up	Adult male patients receiving wound instillation of 1000 mcg of ultra-purified capsaicin (ALGRX 4975, *n* = 20) after open mesh groin hernia repair versus placebo (water, *n* = 21)	The study analysis showed during the first 3–4 days after inguinal hernia repair, capsaicin has superior analgesia compared to placebo [94].
Predel, H.-G. et al.	*Pain and Therapy* 2020	Randomized, double-blind, controlled, multicenter, parallel group trial	746 patients, treated twice daily for 5 days, 6 days follow up	Patients with acute back or neck pain were treated with topical diclofenac 2% + capsaicin 0.075% (*n* = 225), diclofenac 2% (*n* = 223), capsaicin 0.075% (*n* = 223) or placebo (*n* = 75)	Capsaicin alone and capsaicin + diclofenac showed superior benefit compared with placebo. However, diclofenac alone demonstrated efficacy comparable with placebo, and therefore its addition to capsaicin added no increased pain relief over capsaicin alone [95].
Winocur, E. et al.	*Journal of Orofacial Pain* 2000	Randomized, double-blind, placebo-controlled study	30 patients, 4 weeks topical application 4 times daily with follow up at end of each week	Patients with unilateral pain in the temporomandibular joint (TMJ) area received capsaicin 0.025% cream (*n* = 17) or its vehicle (placebo, *n* = 13) to the painful TMJ area	There was no statistically significant difference between the experimental and placebo groups. The factor of time had a major effect on the non-specific improvement of the assessed parameters and the placebo effect had an important role in treating the patients with TMJ pain [114].
McCleane, G.	*British Journal of Clinical Pharmacology* 2000	Randomized, double-blind, placebo-controlled study	200 (151 patients provided results), 4 weeks treatment and follow up	Patients applied topical 3.3% doxepin (*n* = 41), 0.025% capsaicin (*n* = 33), placebo (aqueous cream, *n* = 41), or 3.3% doxepin/ 0.025% capsaicin cream (*n* = 36) thrice daily	Overall pain was substantially reduced by 0.025% capsaicin, 3.3% doxepin and also their combination. The combination of doxepin, and capsaicin resulted in more rapid onset and analgesia. Capsaicin substantially decreased sensitivity and shooting pain [115].
Ellison, N. et al.	*Journal of Clinical Oncology: Official Journal of the American Society of Clinical Oncology* 1997	Placebo-controlled trial	99 patients, four times daily treatment for 16 weeks, weekly questionnaires as follow up	All patients with postsurgical neuropathic pain receiving 8 weeks of the placebo cream versus 8 weeks of 0.075% capsaicin cream	Topical capsaicin cream significantly reduces postsurgical neuropathic pain after the first 8 weeks. It was preferred over placebo by a three-to-one margin [116].
Lazzeri, M. et al.	*The Journal of Urology* 1996	Prospective randomized trial	36 patients, twice weekly treatment for 1 month, 6 months follow up	Patients receiving 10 microM intravesical capsaicin (*n* = 18) or placebo (*n* = 18)	While intravesical instillation of capsaicin was effective on frequency and nocturia in patients with the hypersensitive disorder, its effect on pain score compared to placebo was not confirmed. Possibly higher doses of capsaicin can be effective in pain control and neurological bladder disease [117].
Berger, A. et al.	*Journal of Pain and Symptom Management* 1995	Pilot study	11 patients, 4–6 candies over 2–4 days, 20 min follow ups after each treatment	6 women and five men with oral mucositis pain caused by cancer therapy received oral capsaicin (0.002–0.003 g) in a candy (taffy) vehicle	Oral capsaicin in a candy vehicle resulted in significant pain reduction. The pain relief for most patients was not complete and temporary [118].
Dini, D et al.	*Pain* 1993	Open-label trial	21 (19 evaluable patients), 3 times daily treatment for 2 months, 3 months follow up	Patients with post-mastectomy pain syndrome receiving topical 0.025% capsaicin treatment	Topical capsaicin 0.025% resulted in the disappearance of all symptoms in 2 patients and a significant reduction in 11 patients. Further experimental and clinical research is recommended [119].
**Review Articles**					
Derry, S et al.	*The Cochrane Database of Systematic Reviews* 2017	Systematic review of randomized, double-blind, placebo-controlled studies	2488	Patients with neuropathic pain receiving high-concentration (5% or more) topical capsaicin versus placebo control or 0.04% topical capsaicin as an ‘active’ placebo to help maintain blinding	Patients with HIV neuropathy, postherpetic neuralgia, and painful diabetic neuropathy experienced moderate or substantial pain relief from high-concentration topical capsaicin compared to the control group. High-concentration capsaicin for chronic pain has similar effects to other therapies. These results should be reviewed with caution as the quality of the evidence was moderate or very low [120].
Mou, J. et al.	*The Clinical Journal of Pain* 2014	Meta-analysis of randomized, double-blind, controlled studies	1313	Patients received 8% capsaicin patch (Qutenza) or a control 0.04% capsaicin patch.	Capsaicin 8% patch is effective in a high number of patients suffering from various neuropathic indications, such as postherpetic neuralgia and HIV neuropathy. Its analgesic effect starts within a few days and lasts for an average of 5 months [121].
Laslett, L. L. et al.	*Progress in Drug Research* 2014	Systematic review of five double-blind RCTs and one case-crossover trial of topical capsaicin	-	Topical capsaicin treatment (0.025 to 0.075% formulations) in patients with osteoarthritis versus placebo	Topical capsaicin applied four times daily in patients with clinical or radiologically diagnosed osteoarthritis and at least moderate pain results in moderately effective pain relief up to 20 weeks regardless of the application site and dosage. Topical capsaicin was well tolerated [122].
Mason, L. et al.	*BMJ (Clinical Research Ed.)* 2004	Systematic review, meta-analysis of RCTs	1024	Adults with chronic pain from neuropathic or musculoskeletal conditions receiving topical capsaicin (0.025 to 0.075% formulations) with placebo or another treatment	Topical capsaicin has poor to moderate efficacy in treating musculoskeletal or neuropathic chronic pain. However, it can be used as a sole therapy or an adjunct in patients who are intolerant or unresponsive to other treatments [123].

**Table 6:** Clinical study and review articles on thunder god vine and pain management.

Author	Journal and Year of Publication	Type of Study	Number of Patients, Duration of Study	Study Groups, Active Agent and Placebo Dosage	Key Finding(s)
**Clinical Study**					
Jiao J, et al.	*Journal of Traditional Chinese Medicine* 2019	Double-blinded, randomized, placebo controlled clinical trial	70 patients, 4 weeks of treatment and follow-up	Patients with active rheumatoid arthritis including pain and swelling receiving TwHF cream twice a day (*n* = 35) versus placebo (*n* = 35). The TwHF cream prescription contains TwHF, Chuanxiong, liquid adjuvant Ruxiang and liquid adjuvant Moyao in proportion 4:4:2:2:1; the raw herbs dose is 4 g/mL.	The patients receiving TwHF had improvement of joint tenderness and swelling compared to the placebo group. It can be effective and safe for a patient with active rheumatoid arthritis for short-term use [124].
**Review Articles**					
Li H, et al.	*Clinical Rheumatology* 2015	Systematic review and meta-analysis of RCTs	807	Patients with ankylosing spondylitis (AS) receiving Tripterygium glycosides tablets (an extract of TwHF)	Based on the meta-analysis of the moderate quality clinical trials, Tripterygium glycosides tablets were not effective in treating AS [133].
Cameron, M. et al.	*The Cochrane Database of Systematic Reviews* 2011	Systematic review	23	*Tripterygium wilfordii* to placebo and one to sulfasalazine	While *T. wilfordii* products can reduce some of the rheumatoid arthritis symptoms, its oral use is associated with several mild to moderate adverse effects. The adverse effects resolved after the intervention stopped [127].
Canter, P. H. et al.	*Phytomedicine: International Journal of Phytotherapy and Phytopharmacology* 2006	Systematic review	105	Patients with rheumatoid arthritis receiving *Tripterygium wilfordii* or placebo	While *T. wilfordii* has beneficial effects in reducing rheumatoid arthritis symptoms, due to its association with serious adverse events, we cannot recommend its use [125].

**Table 7:** Clinical studies on butterbur and pain management.

Author	Journal and Year of Publication	Type of Study	Number of Patients, Duration of Study	Study Groups, Active Agent and Placebo Dosage	Key Finding(s)
Pothmann, R. et al.	*Headache* 2005	Multicenter prospective open-label study	108 patients, 4 months of treatment and follow-up	Patients between the ages of 6 and 17, suffering from migraine for at least 1 year. Patients received 50 to 150 mg of the butterbur root extract depending on age for 4 months. Capsules contain extract (drug:extract radio 28:44) with a minimum of 15% petasins and pyrrolizidine alkaloids removed.	The study demonstrated that butterbur root extract is effective and well tolerated migraine prophylaxis in children and teenagers with a low rate of adverse events [144].
Lipton, R. B. et al.	*Neurology* 2004	Three-arm, parallel-group, randomized trial	245 patients, 4 months of treatment and follow-up	Patient with migraine received oral Petasites extract 75 mg (*n* = 77), or 50 mg (*n* = 79), or placebo (*n* = 77) twice a day	Petasites extract 75 mg twice a day is well tolerated and more effective than placebo in preventing migraine. Petasites 50 mg twice a day is not significantly more effective compared to placebo [145].
Diener, H. C. et al.	*European Neurology* 2004	Independent reanalysis of a randomized, placebo-controlled parallel-group study	60 patients, 12 weeks of treatment and follow-up	33 patients treated with two butterbur capsules 25 mg twice a day and 27 patients with placebo	45% of patients receiving butterbur capsules had improvement in migraine frequency of equal or more than 50% compared to 15% of the placebo group. This small study demonstrates butterbur can be effective and well-tolerated for migraine prophylaxis [146].
Grossman, W. et al.	*Alternative Medicine Review: a Journal of Clinical Therapeutic* 2001	Randomized, group-parallel, placebo-controlled, double-blind clinical study	60 patients, 12 weeks of treatment and follow-up	Patients received either Petadolex (a special *Petasites hybridus* extract, each capsule containing 25 mg of a CO_2_ extract of *Petasites hybridus*, *n* = 33) or placebo (*n* = 27) at a dosage of two capsules twice a day	There was a statistically significant decrease in the frequency of migraine attacks in patients receiving Petadolex compared to placebo. It was exceptionally well-tolerated with no adverse events reported. *Petasites hybridus* can be used as a prophylactic treatment for migraines [147].

**Table 8:** Clinical studies and review article on feverfew and pain management.

Author	Journal and Year of Publication	Type of Study	Number of Patients, Duration of Study	Study Groups, Active Agent and Placebo Dosage	Key Finding(s)
**Clinical Studies**					
Cady, R. K. Et al.	*Headache* 2011	Multi-center pilot study	60 patients, 1 month treatment and follow-up	45 patients suffering from migraine received sublingual LipiGesic (containing 3× feverfew and 2× ginger) and 15 received sublingual placebo	In patients who frequently develop mild headaches before a moderate to severe migraine headache, administrating sublingual feverfew/ginger is effective and safe as a first-line abortive treatment. It is generally well-tolerated, with nausea and oral numbness being the most occurring adverse event [150].
Diener, H. C. et al.	*Cephalalgia: an International Journal of Headache* 2005	Randomized, double-blind, placebo-controlled, multicenter, parallel-group study	170 patients, 16 weeks of treament and follow-up	89 patients with migraine received the stable feverfew extract (MIG-99, 6.25 mg t.i.d.) and 81 patients received placebo	Patients receiving MIG-99 had a significant reduction of frequency of migraine attacks per month (1.9 from 4.76 attacks per month) compared to the placebo group. Feverfew extract shows a favorable benefit-risk ratio and is effective [151].
Pfaffenrath, V. et al.	*Cephalalgia: an International Journal of Headache* 2002	Randomized, double-blind, multicenter, controlled trial with an adaptive design	147 patients, 12 weeks of treatment and follow-up	Patients suffering from migraine with and without aura received one of the three dosages of MIG-99 (2.08 mg, *n* = 37; 6.25 mg, *n* = 36; 18.75 mg, *n* = 39 capsules three times a day) or a placebo (*n* = 35)	MIG-99 did not show a significant effect in migraine prophylaxis. Subsequently, the study did not demonstrate a dose-response relationship. In a small subgroup of patients who had at least four attacks during the 28-day baseline period, MIG-99 showed a favorable benefit-risk ratio with a dosage of 6.25 mg MIG-99 capsules per day. Further studies with a larger sample are needed to verify the findings [152].
Pattrick, M. Et al.	*Annals of the Rheumatic Diseases* 1989	Double-blind, placebo-controlled study	40 patients, 6 weeks follow-up	Female patients with symptomatic rheumatoid arthritis received 70- 86 mg dried chopped feverfew (*n* = 20) or placebo capsules (*n* = 20) once daily for six weeks	The study did not show any significant difference between the laboratory or clinical variables of the groups. There is no apparent benefit from oral feverfew for patients with rheumatoid arthritis [153].
**Review Article**					
Wider B. et al.	*The Cochrane Database of Systematic Reviews* 2015	Systemic review of 6 randomized, placebo-controlled, double-blind trials	561	Patients with migraine headaches receiving feverfew or placebo	Only five of the six trials reported on migraine frequency outcome. All studies were either of high or unclear risk of sample size bias. Due to uncommon outcome measures and heterogeneity between the studies in terms of interventions, design, and participants, a pooled analysis of the findings was not possible. Three trials with small sample sizes reported a positive effect of feverfew. Two rigorous trials did not report a significant difference between feverfew and placebo groups. In conclusion, larger rigorous trials using stable feverfew extracts are needed before a firm conclusion can be drawn [154].

**Table 9:** Clinical studies and review article on willow bark and pain management.

Author	Journal and Year of Publication	Type of Study	Number of Patients, Duration of Study	Study Groups, Active Agent and Placebo Dosage	Key Finding(s)
**Clinical Studies**					
Uehleke, B. et al.	*Phytomedicine: International Journal of Phytotherapy and Phytopharmacology* 2013	Observational study	436 patients; 6 months follow-up	Patients suffering from musculoskeletal disorders received aqueous willow bark extract (STW 33-I; drug-extract ratio 16–23:1, 23–26% total salicin, equivalent of 140 mg salicylic alcohol derivate with two tablets) for 6 months. Comedication with other opioids and NSAIDs was allowed	STW 33-I resulted in a significant reduction of pain and it can be used as a long-term basic treatment for musculoskeletal disorders. It was tolerated well and there was no relevant drug interaction with NSAIDs and opioids [161].
Beer, A. M. et al.	*Phytomedicine: International Journal of Phytotherapy and Phytopharmacology* 2008	Open, multicentric observational study with reference treatment	128 patients completed the study. Treatment duration and follow-up were 6 weeks.	90 patients received standardized willow bark extract preparation, 41 standard therapy and 8 patients a combination of the two. Treatment tablet made from 393.24 mg dry extract, containing 60 mg salicin. 1–2 tablets administered twice a day.	The effect of willow bark extract in treating mild or fairly severe coxarthrosis or gonarthrosis is comparable to that of standard therapies without their adverse events [162].
Biegert, C. et al.	*The Journal of Rheumatology* 2004	Two randomized, controlled, double-blind trials with follow up for 6 weeks.	Trial 1: 127 patients Trial 2: 26 patients. Both trials had treatment and follow-up of 6 weeks	Trial 1: Patients with hip or knee osteoarthritis and a WOMAC pain score of at least 30 mm received willow bark extract (240 mg of salicin/day, *n* = 43), or diclofenac (100 mg/day, *n* = 43), or placebo (*n* = 41) Trial 2: patients with active rheumatoid arthritis receive received willow bark extract (corresponding to 240 mg salicin/day, *n* = 13) or placebo (*n* = 13)	There was no statistical difference between the willow bark extract and placebo groups, indicating that willow bark extract has no relevant efficacy in patients with osteoarthritis or rheumatoid arthritis [157].
Chrubasik, S. et al.	*Rheumatology* (Oxford, England) 2001	Open, randomized, post-marketing study	228 patients, 4 weeks of treatment and follow-up	114 patients with low back pain received daily herbal extract containing 240 mg of salicin (Assalix) and 114 received 12.5 mg of the synthetic cyclooxygenase (COX)-2 inhibitor rofecoxib	There was no significant difference in efficacy between both groups at the doses chosen. Few patients in either group used additional conventional treatment and Assalix treatment was less expensive [163].
Schmid, B. et al.	*Phytotherapy Research: PTR* 2001	Randomized placebo-controlled, double blind clinical trial	78 patients, 2 weeks of treatment and follow-up	39 patients with osteoarthritis received willow bark extract (equivalent of 240 mg salicin/day) and 39 patients received placebo	The Western Ontario and McMaster Universities Osteoarthritis Index (WOMAC) pain score decreased by 14% in the willow bark extract group, demonstrating its superiority over placebo. Willow bark extract has a moderate analgesic effect in patients with osteoarthritis and is well tolerated [156].
**Review Article**					
Vlachojannis, J. E. et al.	*Phytotherapy Research: PTR* 2009	Systematic review	669	Patients with musculoskeletal pain received Assalix or ethanolic extract versus placebo or conventional treatment	There is moderate evidence demonstrating the effectiveness of ethanolic willow bark in patients with low back pain. Further studies are needed to evaluate the effectiveness of higher doses than 240 mg daily salicin to treat rheumatoid arthritis and osteoarthritis. There were minor adverse events during treatment [164].
